# Supplementary figures and images for: Allelic composition of carotenoid metabolic genes in 13 founders influences carotenoid composition in juice sac tissues of fruits among Japanese citrus breeding population
Source: PLoS One. 2021 Feb 4;16(2):e0246468. doi: 10.1371/journal.pone.0246468 (PMC7861536; doi:10.1371/journal.pone.0246468)

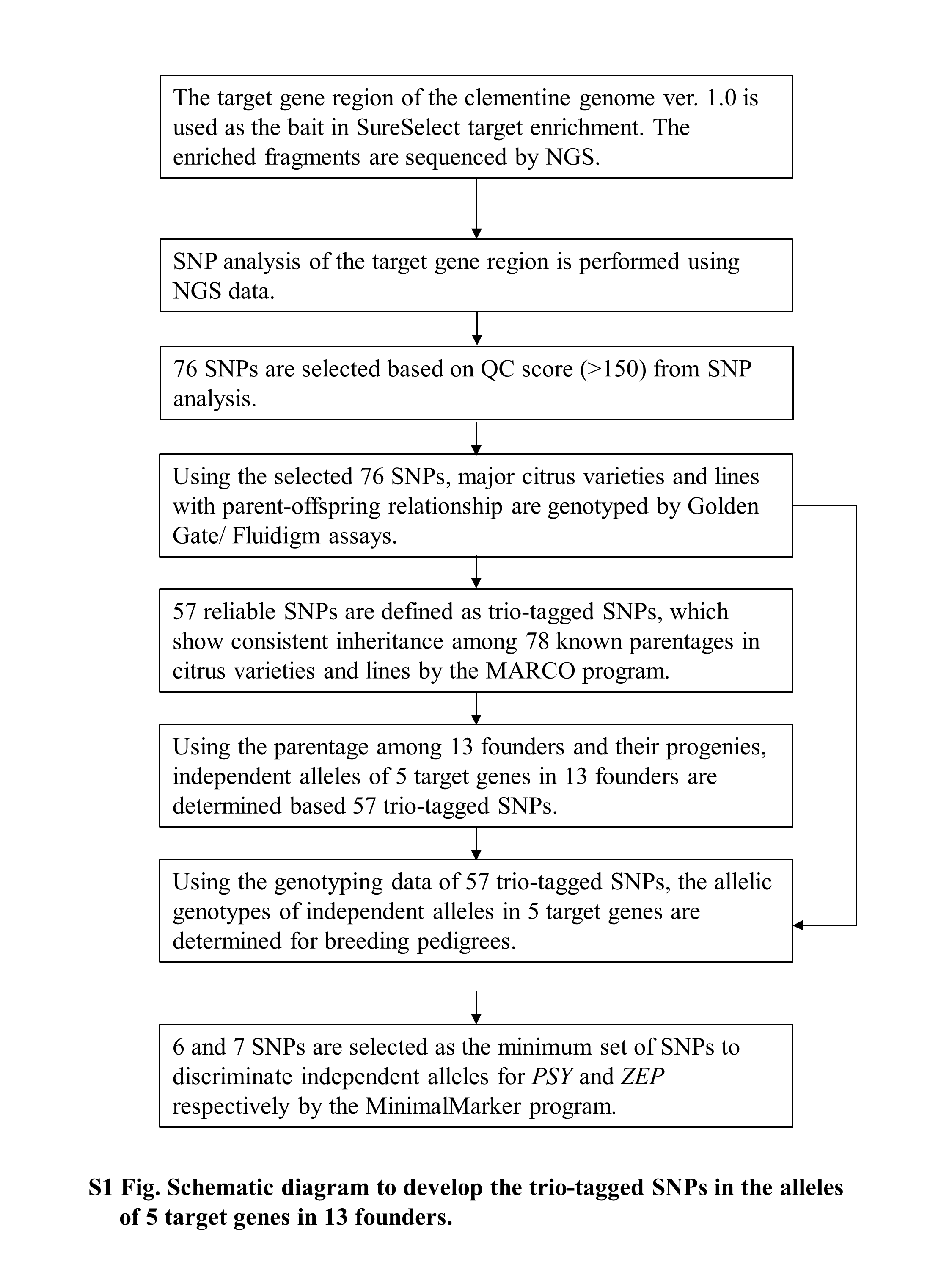

Supplement: S1 Fig — (TIF) [file pone.0246468.s001.tif]

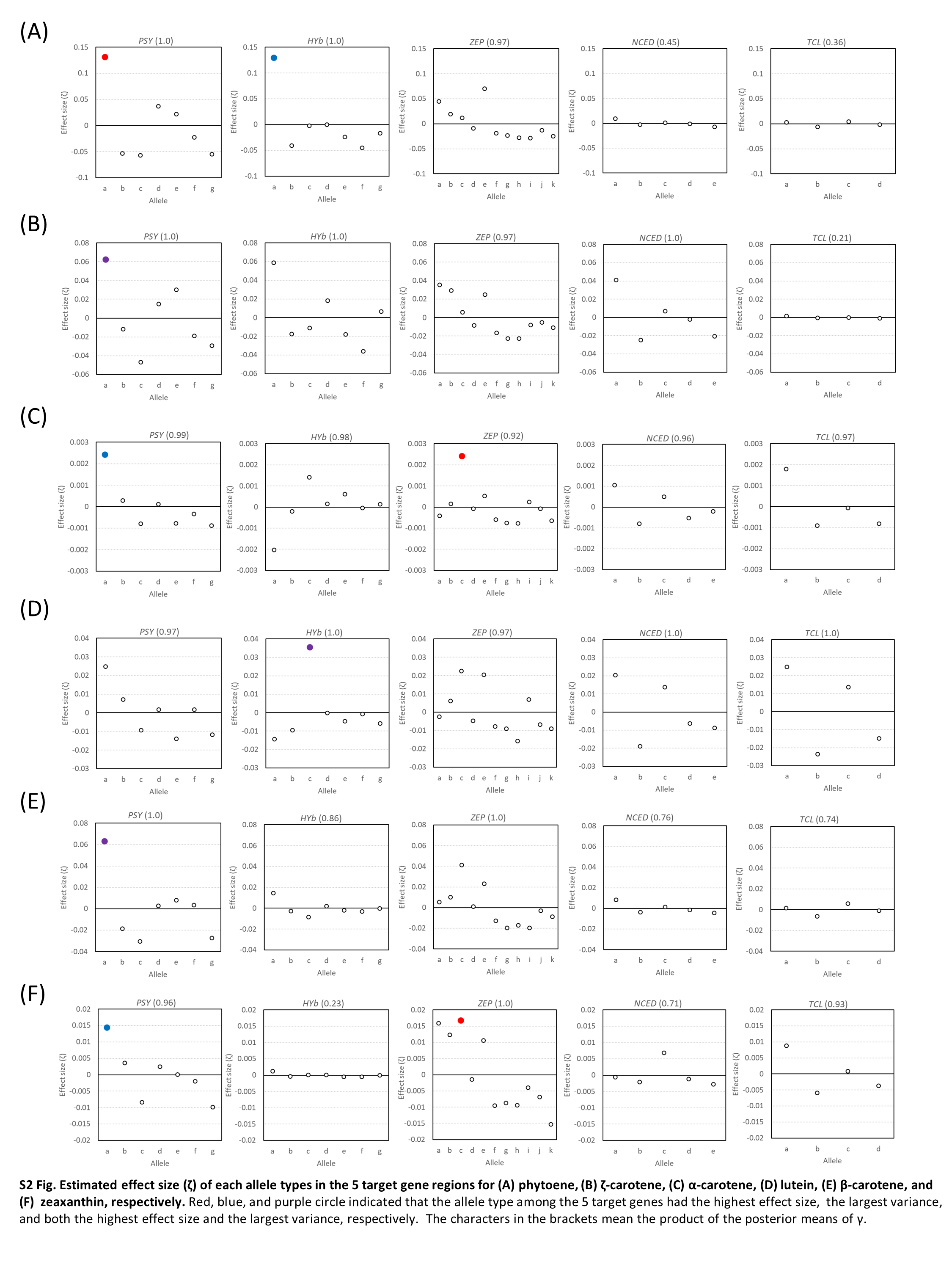

Supplement: S2 Fig — Estimated effect size (ζ) of each allele types in the 5 target genes regions for (A) α- carotene, (B) β-carotene, (C) lutein, (D) phytoene, (E) ζ-carotene, and (F) zeaxanthin, respectively. Red, blue, and purple circle indicated that the allele type among the 5 target genes had the highest effect size, the largest variance, and both the highest effect size and the largest variance, respectively. The characters in the brackets mean the product of the posterior means of γ. (TIF) [file pone.0246468.s002.tif]

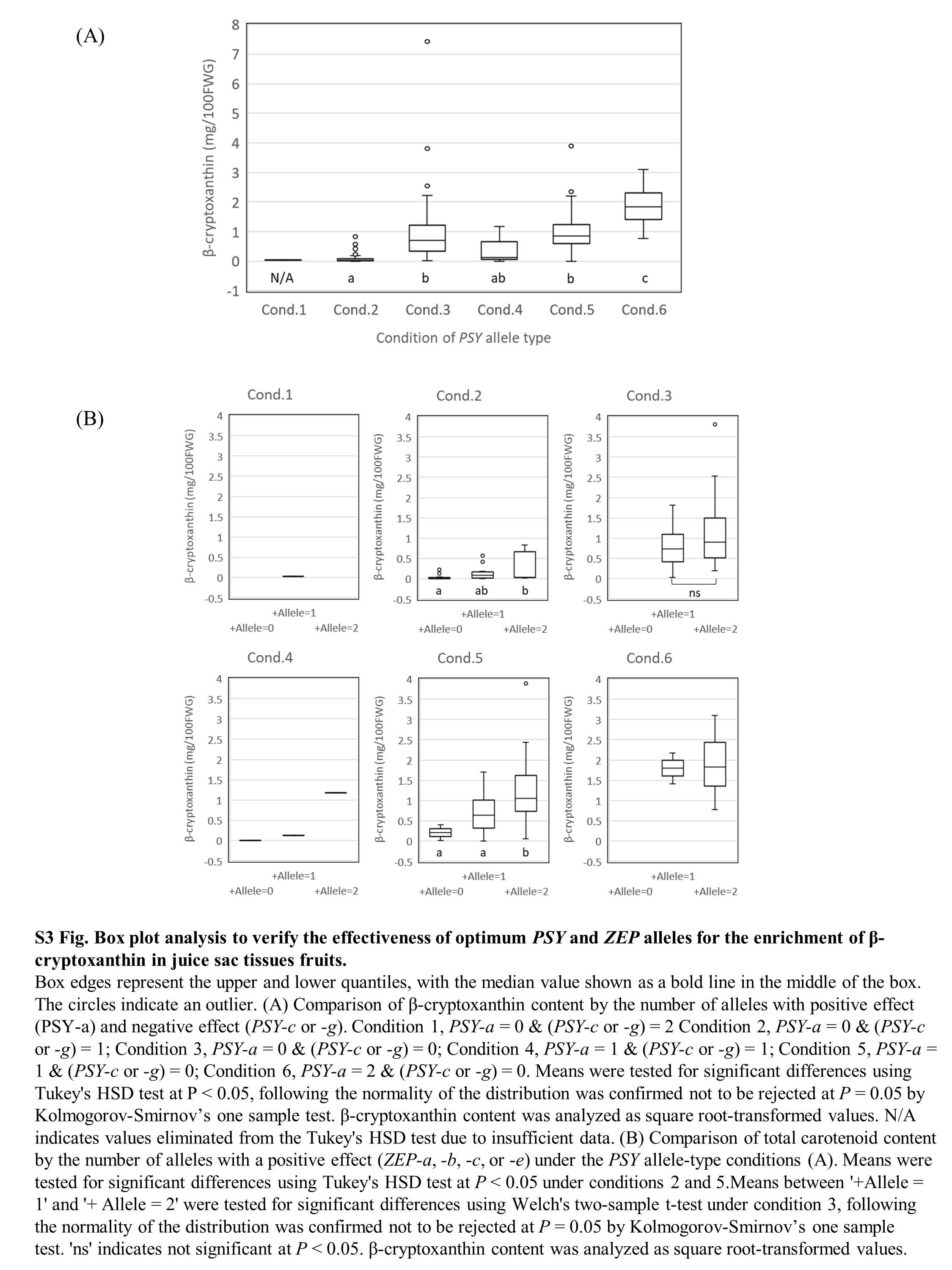

Supplement: S3 Fig — Box edges represent the upper and lower quantiles, with the median value shown as a bold line in the middle of the box. The circles indicate an outlier. (A) Comparison of β-cryptoxanthin content by the number of alleles with positive effect (PSY-a) and negative effect (PSY-c or -g). Condition 1, PSY-a = 0 & (PSY-c or -g) = 2 Condition 2, PSY-a = 0 & (PSY-c or -g) = 1; Condition 3, PSY-a = 0 & (PSY-c or -g) = 0; Condition 4, PSY-a = 1 & (PSY-c or -g) = 1; Condition 5, PSY-a = 1 & (PSY-c or -g) = 0; Condition 6, PSY-a = 2 & (PSY-c or -g) = 0. Means were tested for significant differences using Tukey’s HSD test at P < 0.05, following the normality of the distribution was confirmed not to be rejected at P = 0.05 by Kolmogorov-Smirnov’s one sample test. β-cryptoxanthin content was analyzed as square root-transformed values. N/A indicates values eliminated from the Tukey’s HSD test due to insufficient data. (B) Comparison of total carotenoid content by the number of alleles with a positive effect (ZEP-a, -b, -c, or -e) under the PSY allele-type conditions (A). Means were tested for significant differences using Tukey’s HSD test at P < 0.05 under conditions 2 and 5. Means between ’+Allele = 1’ and ’+ Allele = 2’ were tested for significant differences using Welch’s two-sample t-test under condition 3, following the normality of the distribution was confirmed not to be rejected at P = 0.05 by Kolmogorov-Smirnov’s one sample test. ’ns’ indicates not significant at P < 0.05. β-cryptoxanthin content was analyzed as square root-transformed values. (TIF) [file pone.0246468.s003.tif]

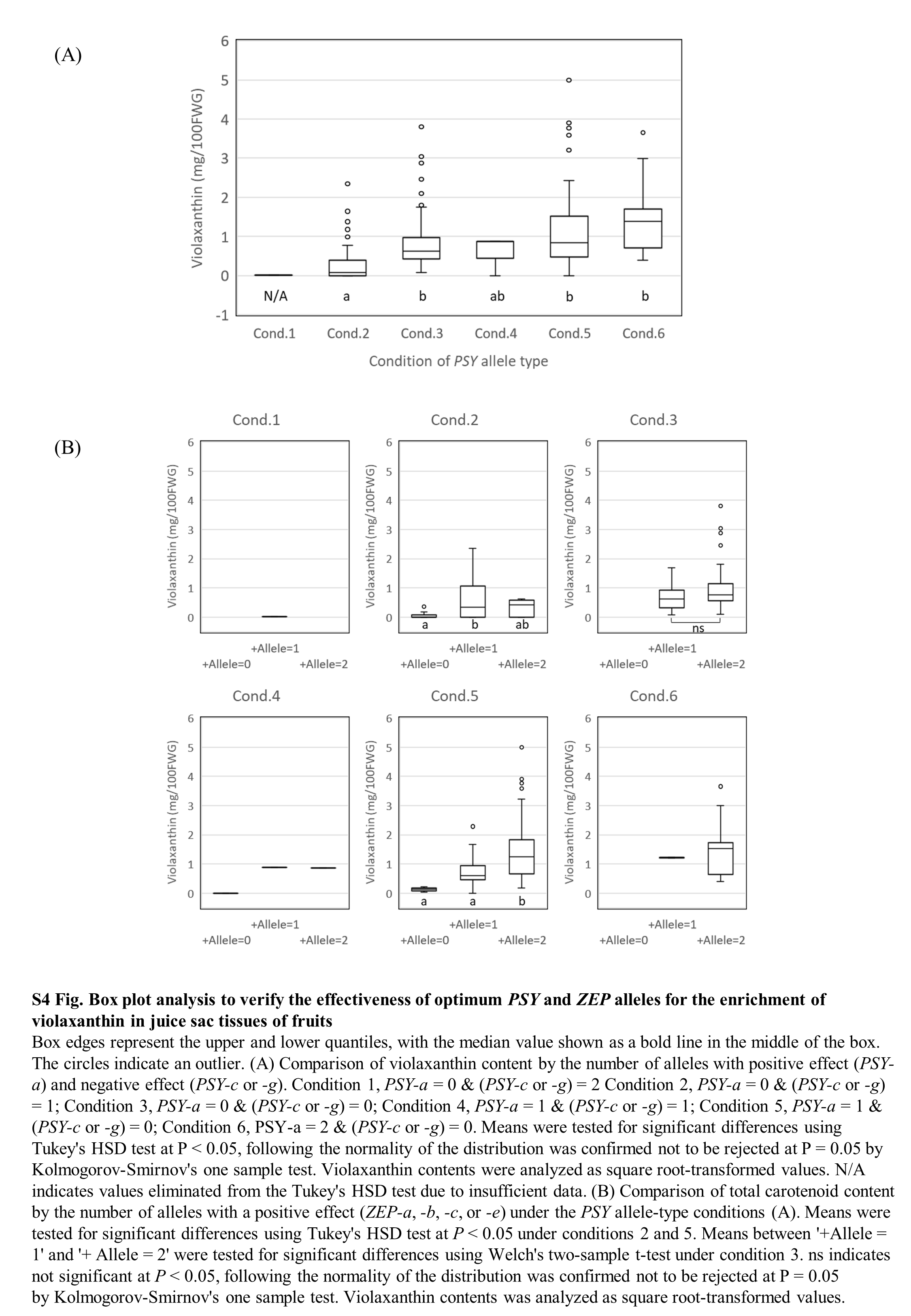

Supplement: S4 Fig — Box edges represent the upper and lower quantiles, with the median value shown as a bold line in the middle of the box. The circles indicate an outlier. (A) Comparison of violaxanthin content by the number of alleles with positive effect (PSY-a) and negative effect (PSY-c or -g). Condition 1, PSY-a = 0 & (PSY-c or -g) = 2 Condition 2, PSY-a = 0 & (PSY-c or -g) = 1; Condition 3, PSY-a = 0 & (PSY-c or -g) = 0; Condition 4, PSY-a = 1 & (PSY-c or -g) = 1; Condition 5, PSY-a = 1 & (PSY-c or -g) = 0; Condition 6, PSY-a = 2 & (PSY-c or -g) = 0. Means were tested for significant differences using Tukey’s HSD test at P < 0.05, following the normality of the distribution was confirmed not to be rejected at P = 0.05 by Kolmogorov-Smirnov’s one sample test. Violaxanthin contents were analyzed as square root-transformed values. N/A indicates values eliminated from the Tukey’s HSD test due to insufficient data. (B) Comparison of total carotenoid content by the number of alleles with a positive effect (ZEP-a, -b, -c, or -e) under the PSY allele-type conditions (A). Means were tested for significant differences using Tukey’s HSD test at P < 0.05 under conditions 2 and 5. Means between ’+Allele = 1’ and ’+ Allele = 2’ were tested for significant differences using Welch’s two-sample t-test under condition 3. ns indicates not significant at P < 0.05, following the normality of the distribution was confirmed not to be rejected at P = 0.05 by Kolmogorov-Smirnov’s one sample test. Violaxanthin contents was analyzed as square root-transformed values. (TIF) [file pone.0246468.s004.tif]

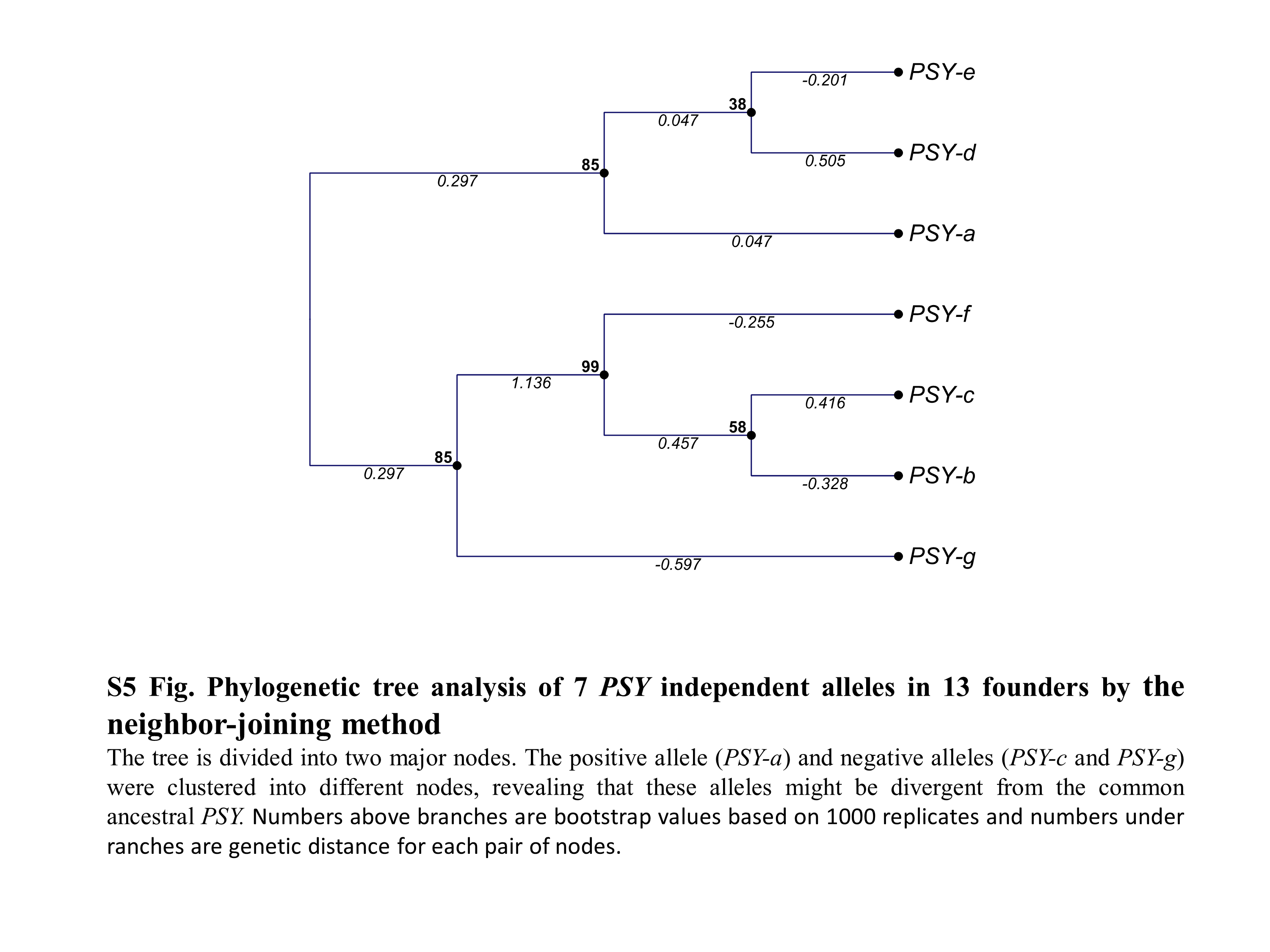

Supplement: S5 Fig — The tree is divided into two major nodes. The positive allele (PSY-a) and negative alleles (PSY-c and PSY-g) were clustered into different nodes, revealing that these alleles might be divergent from the common ancestral PSY. Numbers above branches are bootstrap values based on 1000 replicates and numbers under ranches are genetic distance for each pair of nodes. (TIF) [file pone.0246468.s005.tif]

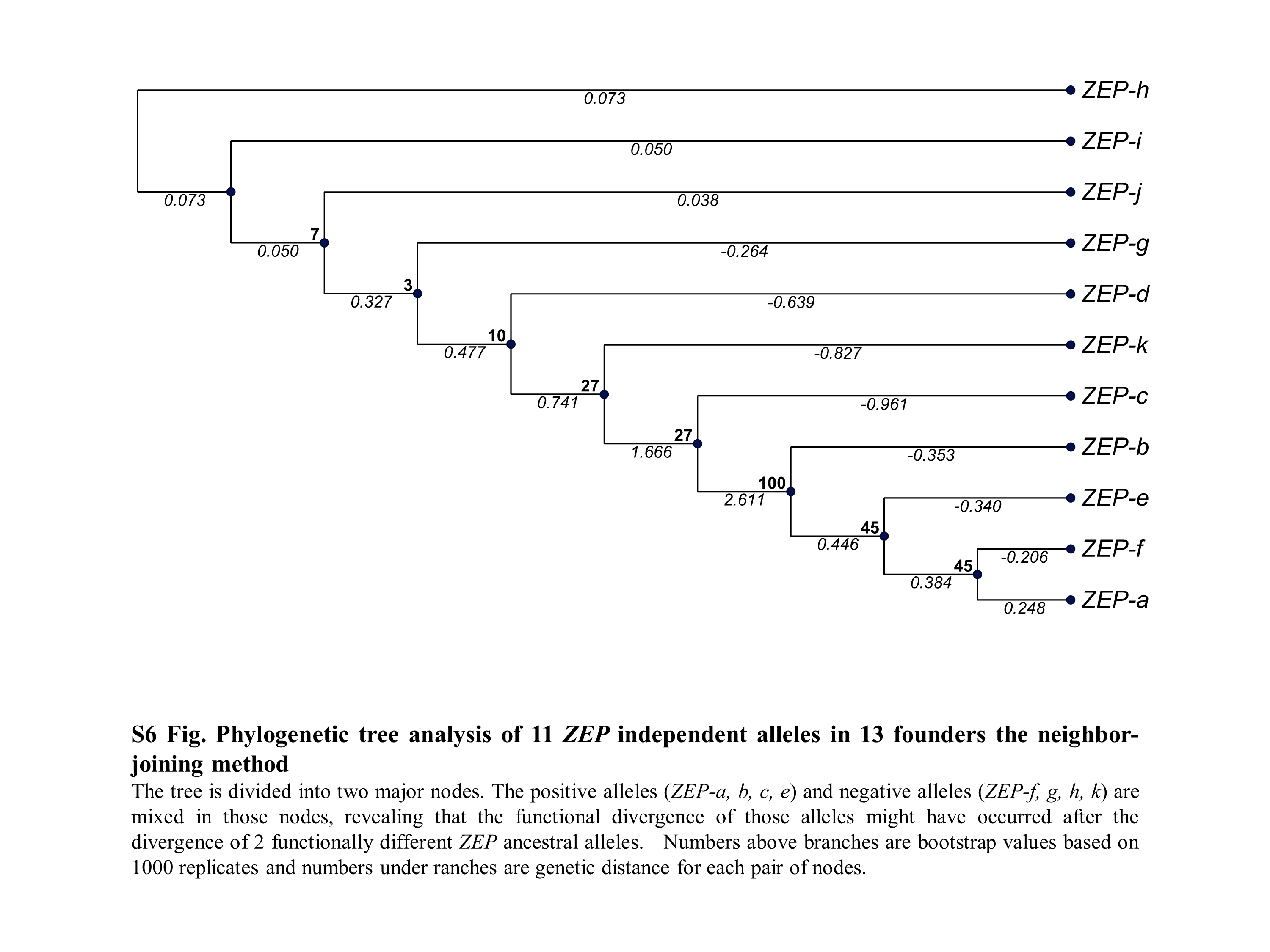

Supplement: S6 Fig — The tree is divided into two major nodes. The positive alleles (ZEP-a, b, c, e) and negative alleles (ZEP-f, g, h, k) are mixed in those nodes, revealing that the functional divergence of those alleles might have occurred after the divergence of 2 functionally different ZEP ancestral alleles. Numbers above branches are bootstrap values based on 1000 replicates and numbers under ranches are genetic distance for each pair of nodes. (TIF) [file pone.0246468.s006.tif]
