# Supplementary material for: Allelic composition of carotenoid metabolic genes in 13 founders influences carotenoid composition in juice sac tissues of fruits among Japanese citrus breeding population
Source: PLoS One. 2021 Feb 4;16(2):e0246468. doi: 10.1371/journal.pone.0246468 (PMC7861536; doi:10.1371/journal.pone.0246468)
Supplement: S1 Table — (PDF) [file pone.0246468.s007.pdf]

S1 Table. Information on reliable trio-tagged SNPs with lineage by trio-analysis

SNP name	Scaffold	SNP position in clementine genome v1.0	Gene	5'UTR (5) / CDS (C) / Intron (I) / 3'UTR (3)	Synonymous (S) / Different (D) replacement	Amino acid position in gene	Amino acid in refference gene	Aaltanavive amino acid in refference gene	Strand of gene	SNP position in gene	SNP reference base in refference gene	SNP altanavive base in refference gene	Periferal genomic sequence of SNP	SNP detection : GoldenGate (GG) / Fluidigm (FD)
PSY-SNP01	6	21,395,732	Ciclev10011841m.g	I					-	-171	A	G	CTCATTCATAGCATATCCTTTTAGCTTTCTT[A/G]AAATAACTCACCCCATGGTTTCTCTCTTTTTGCTCCTTACTAATAATGCTGTGCTGAGAGTTCTCTGACAAGCTTTCTCTGATTTCTGGTATCAGGATTCAG	GG
PSY-SNP02	6	21,394,299	Ciclev10011841m.g	I					-	1263	G	A	CTATGGATAATTCACCCCTAAGTTGCACCTCTTTATTACAATAAGGTTCCATCAAGAGAAATGATCAACACCAACC[G/A]GGATGTTGTATCATTAGCATTTTGAATCTCATCTGTACAAGGTGGTGAATGTTTGTTTCCCATAATTTTGGCT	GG
PSY-SNP03	6	21,394,190	Ciclev10011841m.g	I					-	1372	A	T	GAATCTCATCTGTACAAGGTGGTGAATGTTTGTTTCCCATAATTTTGGCTAGCAGGATTGTTTTGTTGTCTGGGCAGCAGGTCAT[A/T]CTAGTAAGAGAACAATTGGAAGAGTAATTCATTAAGGTATAAGTTTAATTTAATCACATATAGT	GG/FD
PSY-SNP04	6	21,393,579	Ciclev10011841m.g	I					-	1983	T	C	GAAATCTATCTATTCTGAATGCTTGGAGTCTCGTTGCAAATTCTTTATTAGTCATCTCTCCTCCAAACATATTCCTTCTATCCTTTCTTTCCTTTGTTAC[T/C]CTTAATATGTTTGTTGTGTGAGCAATTTCTGTTGCTGCATGAAAACCATACATTTAAAAACATATATTTCCTTTCTCTATTTAAAACATACATTCCTTTC	GG/FD
PSY-SNP05	6	21,393,414	Ciclev10011841m.g	I					-	2148	T	C	TATTTCCTTTCTCTATTTAAAACATACATTCCTTTCTCTAAAAAAAAAAAAAACATTTAAAAACATATATTTCCGTTCTCTATTGTTTCTCATGTTTTCT[T/C]CAATTTCAGTGTGGTGTAGGAGGACAGATGAGCTCGTTGATGGGCCTAATGCTTCACACATAACTCCAACAGCTTTAGAAAGGTGGGAGTCCAGGTTGGA	GG
PSY-SNP06	6	21,393,093	Ciclev10011841m.g	I					-	2469	G	A	AAATGGTTGTTATAGTACTCTCTCTACTATCTTAAGTGTACTTGTATTAAATTAAAATAAGGAACAACTTCTGCTTTCTAATTGGTTTTTAAAACATTAA[G/A]CCTTGATGCATAATGACAGACCTTATTTACATTTAATTGAGTCATGCCATTTTTGCATTTTCAATTTATCCTAGAGACCGAAGATGTGATGAGGTGACGC	GG
PSY-SNP07	6	21,392,826	Ciclev10011841m.g	I					-	2736	A	C	TAACGGATAGCAGTAGTGAATTTACTTATCTGAAAACAAAGTTCTGCATGCTACCCTTCTCAATATTCAGACAAGAGTTTAATAGGCCTGCGATATCTAA[A/C]TAAAGGATGCAGTTTCTGACTGAACCACCTCCCTGCAACGTTATCTTTTGTACCTTGATCTTTCTTCAGAAAATGTTCTATTAAAAGTATTTCCAGTGGA	GG/FD
PSY-SNP08	6	21,392,589	Ciclev10011841m.g	I					-	2953	G	A	TAAGATACATAAACTTATTGGTCATCCCTAATTGAAGGTTGTACCTTTAGGATCTGTTTATTCTGCAATGCTAGTATTGAACTTGTCAAAGATTTATTGA[G/A]CTCTTTTTTGTTTTTGCAGCCATTCAGAGATATGATAGAAGGAATGAGGATGGACCTTAGGAAGTCAAGATACAAAAACTTTGATGAATTATACTTGTAT	GG
PSY-SNP09	6	21,392,572	Ciclev10011841m.g	I					-	2990	C	T	TTGGTCATCCCTAATTGAAGGTTGTACCTTTAGGATCTGTTTATTCTGCAATGCTAGTATTGAACTTGTCAAAGATTTATTGAGCTCTTTTTTGTTTTTG[C/T]AGCCATTCAGAGATATGATAGAAGGAATGAGGATGGACCTTAGGAAGTCAAGATACAAAAACTTTGATGAATTATACTTGTATTGTTATTATGTTGCTGG	FD
PSY-SNP10	6	21,392,073	Ciclev10011841m.g	I					-	3489	T	C	TCAGTCAAAAGTATGTTTTAATTTAATGATCATTTCAATTTGAACCAATCAGTACATTGTTTGGAAAGCCAGGATGCCCTTAACCAGAATTTTGGTTTGC[T/C]GACTGCATGAGGCTCCAGTTGTTTGACATCTGATTTATTTACAATACTATATATTCTCCGTTTGTAGAAGGTTAGTTAATCAGGTCCTGACGGTCATAAA	FD
PSY-SNP11	6	21,391,041	Ciclev10011841m.g	3					-	4521	G	A	AAAAATGAAAATGATTGCTTGTATATTAGGAATTGTTGGTATGCAGCATGTATTTGATGGTAAAGTTAGAATAGTGAATCCAATTCACAATCCAAGGCCG[G/A]TGCCCTATTCAATATTGTAGATTCATTATATTGTCCTCCCAAGATTATACCGTGCAACAATGTGGCCCGTATAAAATTAATCCTCCAAGATTAACGAAGT	GG
PSY-SNP12	6	21,390,869	Ciclev10011841m.g	3					-	4693	G	T	TAAAATTAATCCTCCAAGATTAACGAAGTGAAATAGTAAAAAACGCAAAACTCAATAATGAGAACAGAGGGTGTGGTAACCATATAAATTTGTCAAGCCA[G/T]AATGCATACAAGAGTTTGTATGAATTGCCTGTACAAGTAACCCTGTAACAGTATAATCATAATGTAAAGACAAGTCTATCAACGTATTTATAATGGTGTT	FD
HYb-SNP01	9	29,491,151	Ciclev10005481m.g	5					-	-169	A	G	GTATGTATTTTATTCCCCAAAGCTTT[A/G]CAGGAGAAATTGTGGTCTGCGTCCATTTAACAACCACACAAGAAGTCCAGAATTTCACGCTCACGCCATTAAACACCACAATCCACAATCCACT	GG
HYb-SNP02	9	29,490,958	Ciclev10005481m.g	C	D	9	I	V	-	25	A	G	TCACATCAACTCTTCCTCTTTTCAAGTGCTTTTACTCTAAAACCCAAAACCTCGTAAACAAACAAAACCCCACCATGGCGGTCGGACTATTGGCCGCC[A/G]TAGTCCCGAAGCCCTTCTGTCTCCTCACAACAAAACTTCAACCCTCTTCGCTCCTCACAACAAAACC	GG
HYb-SNP03	9	29,490,536	Ciclev10005481m.g	I					-	447	G	A	ATTACAGGTTCTGGTGGCAAATGGAGGTACTTCAAACAAATCACACATGTCCTAATGTTATTGGTTGGTT[G/A]TATGAACAGAAAATTTCGCCCCTCTTTTGATGATGCTTACATGTTATGTATCCGTACAGGGTGGAGAGGTGCCTTTAGC	GG/FD
HYb-SNP04	9	29,490,045	Ciclev10005481m.g	I					-	938	G	C	GGTCTCAATATTTTTTTGGGGAAGTTATAACATGTGATGTATGTAATTGAAATTAAAAAAA[G/C]AAAGTTATTTTCGGAATCTCATTGCAGTCTCACCATCGACCAAGAGAGGGTCCTTTTGAGCTAAACGATGTGTTTGCCATAATCAACG	GG
HYb-SNP05	9	29,489,930	Ciclev10005481m.g	C	D	208	F	V	-	1053	T	G	AATCTCATTGCAGTCTCACCATCGACCAAGAGAGGGTCCTTTTGAGCTAAACGATGTGTTTGCCATAATCAACGCAGTTCCAGCCATAGCCCTTCTCTCT[T/G]TTGGCTTCTTCCACAAAGGCCTTGTACCTGGTCTCTGTTTTGGTGCTGTAAGTTCTCAATAATCCCATAACTAATAAAATTAAATTAATTCCCCGAATTA	GG
HYb-SNP06	9	29,489,892	Ciclev10005481m.g	C	S	220	C	C	-	1091	T	C	CTTTTGAGCTAAACGATGTGTTTGCCATAATCAACGCAGTTCCAGCCATAGCCCTTCTCTCTTTTGGCTTCTTCCACAAAGGCCTTGTACCTGGTCTCTG[T/C]TTTGGTGCTGTAAGTTCTCAATAATCCCATAACTAATAAAATTAAATTAATTCCCCGAATTAGCGGCGGGATTTGTTAATTAATTAGTTAGTTACTTAAC	GG/FD
HYb-SNP07	9	29,489,840	Ciclev10005481m.g	I					-	1143	T	C	CCTTCTCTCTTTTGGCTTCTTCCACAAAGGCCTTGTACCTGGTCTCTGTTTTGGTGCTGTAAGTTCTCAATAATCCCATAACTAATAAAATTAAATTAAT[T/C]CCCCGAATTAGCGGCGGGATTTGTTAATTAATTAGTTAGTTACTTAACGGGCGCATGATTCTGGGGGAATCAGGGACTTGGCATTACGGTGTTTGGGAT	GG
HYb-SNP08	9	29,489,649	Ciclev10005481m.g	C	S	262	A	A	-	1334	G	T	TGTTTGGGATGGCCTACATGTTCGTCCACGATGGTCTCGTTCACAAAAGGTTCCCTGTGGGTCCCATTGCCGACGTGCCTTATTTCCGGAGAGTCGCTGC[G/T]GCTCACCAGGTAAATGACCAAATTATGAATTAATTACGCACTAATAAACCGTTTTCAGTTATTTTTAACAAAATTAAATGAAAACTCTCTCTTTACGGGA	GG/FD
HYb-SNP09	9	29,488,937	Ciclev10005481m.g	C	D	309	V	I	-	2046	G	A	GCTTGAAGAAGTGGGGGGACTAGAAGAATTGGAGAAGGAGATCAGTAAGAGAATCAAATCATACAACAGG[G/A]TTCCAAAATAATCAATTTAATGGGAGGACCAATTTTTGGATCAATTTGTCAGTGTA	GG/FD
HYb-SNP10	9	29,488,880	Ciclev10005481m.g	3					-	2103	C	G	TTCCAAAATAATCAATTTAATGGGAGGACCAATTTTTGGATCAATTTGTCAGTGTA[C/G]AGAAACAATAGTGTTATTAATGAAAAAAATAAATTATGAATGCTTATGGGTGGATTACTGTTGTAAAGTTTA	GG/FD
ZEP-SNP01	7	3,228,529	Ciclev10025089m.g	C	D	33	E	D	-	99	A	T	AGATGGTTTCATCTATGTTCTACAATTCAGTCAATCTTTCAACAGCTGTTTTCTCAAGAACCCACTTCCCAGTTCCTGTATACAAACATTCTTGCATTGA[A/T]TTTTCACGTTATGATCACTGTATTAACTACAAATTCAGGACCGGAACAAGTGGGCAAAGCAAGAACCCGACACAAATGAAAGCTGCAGTGGCAGAGTCCC	GG

ZEP-SNP02	7	3,228,457	Ciclev10025089m.g	C	S	57	T	T	-	171	A	G	TTTTCACGTTATGATCACTGTATTAACTACAAATTCAGGACCGGAACAAGTGGGCAAAGCAAGAACCCGAC[A/G]CAAATGAAAGCTGCAGTGGCAGAGTCCCCTACTAATAACAGTGACAGTGAAAACAAGAAATTGAGGATTTTGGTGGCTGGT	GG
ZEP-SNP03	7	3,228,016	Ciclev10025089m.g	I					-	612	C	A	TGAATCTCCCCTGGTGTGTTTAACTAAGTAGTCCACAAATTCTGATAGACTTTATGTTCTATTGGAGAT[C/A]TTATGAATGAGAGAACGGAGTCAATTGCCAATGACATGGAGATGGGTGCGTATTTTATGTACTTCGTGGT	GG
ZEP-SNP04	7	3,227,856	Ciclev10025089m.g	I					-	772	C	G	TACTTCGTGGTACAAGTAGTACAATTGTTATTATGCTAATCTTTTTTTGGACATAGGATCATACTTTTTCTTGTCTATAGTTAAATTGATTCTCAGAACA[C/G]TATAACTACGAGTTTTAGATTGGGAAATAGTCTCTGGTAGTGGACTTTTCTCACATCTGTAGACGCAATTAGCCAAGAACAATATAAAAAAAAAAATCTT	GG
ZEP-SNP05	7	3,227,577	Ciclev10025089m.g	C	S	196	L	L	-	1051	A	T	CCAGTCACAAGAGTAATTAGCCGAATGACTTTGCAACAAATACT[A/T]GCTAAAGCGGTTGGGGATGAAATCATTTTGAATGAAAGTAATGTTATTGATTTTAAGGATCATGGAGATAAGGTAATTG	GG
ZEP-SNP06	7	3,227,488	Ciclev10025089m.g	I					-	1140	T	C	CAACAAATACTAGCTAAAGCGGTTGGGGATGAAATCATTTTGAATGAAAGTAATGTTATTGATTTTAAGGATCATGGAGATAAGGTAATTGAGCTGCTGA[T/C]TGGGTGAAGATCATCTGCCTCACAGATTTCTTTGCTGGTTTAAATTTGCACATTTATGGTTTTGTATTTATGTTATTTATGAGTTCAGGTCTCCGTAGTG	GG
ZEP-SNP07	7	3,227,351	Ciclev10025089m.g	C	D	237	I	V	-	1277	A	G	TAAATTTGCACATTTATGGTTTTGTATTTATGTTATTTATGAGTTCAGGTCTCCGTAGTGCTTGAGAATGGACAGTGTTATGCTGGTGATCTTCTC[A/G]TTGGAGCTGATGGAATATGGTCCAAGGTATCATTCTATGTAATTTTCATTGTGTG	GG
ZEP-SNP08	7	3,226,754	Ciclev10025089m.g	I					-	1874	A	T	AACGCTTAATAAAATTAATTTATGATCTTCTAGGAATTTTTGAATTAAAGAAGATGAAAATAAAAAA[A/T]ATGTCCCTTTTTTAACCCTTATTCCCTTTTTATATTCTATTAAAGTTTCTCCTGTTC	GG
ZEP-SNP09	7	3,226,262	Ciclev10025089m.g	I					-	2366	G	T	AGAACAGCTGGCACTCCATGTTGGGAAAAATTTTGTTCCCAAAATCAAGAAGCTAACAAACGCTCAAAAGTTGAAAATAATGATGCTTCTTCACATTCAA[G/T]AATCTATTCCTTGATTCATTTCATATTTGGCAGGTAAAAAGGAAAGGTTGCTGAAAATATTTGAGGGTTGGTGTGATAATGTGGTAGATTTGATACTTGC	GG/FD
ZEP-SNP10	7	3,225,613	Ciclev10025089m.g	I					-	3015	C	T	ATTCATTCTTTTTGTTGTATTGTTCATCTTTTTTAAATGATATTGACATCAAATTCAGCATGCCGTGGCTATTGTGGTTGTTGTTTTTGTTTTTGCTGTG[C/T]GCGTGGTTGGGGGTAGAAAATATTTTGTTTCTAGTTATCCTTGTTCTAACACCTGATAGCTATCTATTGCCTTTAAGGCTATTGGCATTAGAAATTGGAA	GG
ZEP-SNP11	7	3,225,508	Ciclev10025089m.g	I					-	3120	T	C	GGTTGGGGGTAGAAAATATTTTGTTTCTAGTTATCCTTGTTCTAACACCTGATAGCTATCTATTGCCTTTAAGGCTATTGGCATTAGAAATTGGAAATCA[T/C]GTTTTTATTACTGATTTTCTTTTCTTTTTCTTGATATCAAGAATTAAGATCATTCCCATTATGAATTTGGGGGTACCCCTTCATGGACTTCCACATGTGA	GG
ZEP-SNP12	7	3,225,360	Ciclev10025089m.g	I					-	3268	A	G	TTTTTTTTCTTTCAAATTTGCAGTAGTGTGATAGTTGATGTTTC[A/G]TGAACCATATTTATTATTCAAAATATAATTTCTGTCTAGGCTTTTTGAGATGGGAGAACTGG	GG
ZEP-SNP13	7	3,225,098	Ciclev10025089m.g	C	S	407	S	S	-	3530	A	C	TTCTTTGTTACAGGATGGTTATCAACTTGCAGTAGAGCTTGAGAAAGCCTGTAAAAAAAGCAATGAGTC[A/C]AAAACCCCCATTGACATTGTTTCTGCTCTAAAGAGGTAACAGATGATTTGTTTGCCAAAAAATCTGAAAGAGAAGTGTTGACGTGCCTAGGACCAAAAAA	GG/FD
ZEP-SNP14	7	3,224,776	Ciclev10025089m.g	C	S	438	A	A	-	3852	C	T	GTTTACAACCAACTCTCATTGGTCGTGCTTTTAAATTCTGCAGCTATGAGAGAGCTAGGAGACTGCGAGTGGCTGTTATCCATGGACTGGCAAGATCGGC[C/T]GCAGTGATGGCTTCAACTTACAAGGCTTATTTAGGTGTTGGACTTGGACCGTTGTCGGTGGTTATTTTGGTTATTATCCAATTACTAAAATAATAACTT	GG
ZEP-SNP15	7	3,224,504	Ciclev10025089m.g	I					-	4124	C	T	CCTCATCCAGGAAGAGTAGGTGGTCGATTTTTCATTGACTTAGCAATGCCCTTAATGCTTAGTTGGGTCTTAGGTGGCAACAGGTTAGATTTGGTAGACT[C/T]TGTCCTCTTCTGCATTCTAGCTTTTATCTTTATATTTCACCATCATTAATTTTATGGCAGAACACCTTTGATCCCGAAAACTCAAATCAATGATTTTTAT	GG
ZEP-SNP16	7	3,224,255	Ciclev10025089m.g	I					-	4373	C	T	GTAATCTCATACGACCATATATGCATCAGGTATTATTTAGCTTTTCATGTTTGGAATTCTTTGTGCTTATTGCATTTACTCTAGAATAGCCTTGTTTCTC[C/T]AACACCATAGTAACTCAATGCATTCTCAGTGTTTTTAGGAAGCTCTGGGCCAAGGTTTAAAGTAGGATCCTAAATACTAAAGCATTTAAGTTGGAAAAGA	GG
ZEP-SNP17	7	3,224,192	Ciclev10025089m.g	I					-	4436	T	C	TGCTTATTGCATTTACTCTAGAATAGCCTTGTTTCTCCAACACCATAGTAACTCAATGCATTCTCAGTGTTTTTAGGAAGCTCTGGGCCAAGGTTTAAAG[T/C]AGGATCCTAAATACTAAAGCATTTAAGTTGGAAAAGAAATATCATAAAGATGGATTTCTAAAAATATGCTAAAGCATTCACAAATTTATTTAGTTAAAGG	GG
ZEP-SNP18	7	3,223,842	Ciclev10025089m.g	I					-	4786	T	C	TAATTTCTTCAGGCAAGTGACAACTTACGAACATGGTTTCGTGACGATGACGCATTGGAGCGTGCTATGAATGGAGAGTGCGTATGATAAACCTTATGTT[T/C]GTGAATATCAACCCTTGTGCCTTTTCTAATTCTTGTACACATTCTGAAATCACTGTTTCCATACCCTCTTGTTTATTGTGTAGGTGGTTTCTAGTACCAT	GG
ZEP-SNP19	7	3,223,699	Ciclev10025089m.g	C	D	549	G	V	-	4929	G	T	TCTGAAATCACTGTTTCCATACCCTCTTGTTTATTGTGTAGGTGGTTTCTAGTACCATCTGGAAGTGAGAATGTTGTTTCGCAGCCTATTTATTTAAGTG[G/T]ATCACATGAGAATGAACCCTATTTAATTGGGTATGATTATGATTGTGAATCCGTATTGCTATGAATTATATTTACGTAAATTAATGGCTGATATTTATG	GG/FD
ZEP-SNP20	7	3,223,615	Ciclev10025089m.g	I					-	5013	A	C	TCACATGAGAATGAACCCTATTTAATTGGGTATGATTATGATTGTGAATCCGTATTGCTATTGAATTATATTTACGTAAATT[A/C]ATGGCTGATATTTATGCTATTCAATTAGATTAAGATGGTAATCTATCATATTGATGTGTTATTATGA	GG
ZEP-SNP21	7	3,223,519	Ciclev10025089m.g	C	D	567	S	P	-	5109	T	C	AATTAATGGCTGATATTTATGCTATTCAATTAGATTAAGATGGTAATCTATCATATTGATGTGTTATTATGACTGCAGAAGTGAAAGTCACGAGGATTTT[T/C]CTCGAACATCGATTGTGATCCCTTCAGCGCAGGTGAATTATTGGCTGCTCACCTTGTTTGCCTCGTTGATCTGGGATATAAATAATTAATTTCTGAAGTC	GG
ZEP-SNP22	7	3,223,199	Ciclev10025089m.g	I					-	5429	C	A	GCCAATCTTCAATACTTGGTAGAGCTCTTCTCTAAGATTTTTCCCTTT[C/A]CAAGTGCAGCAATGAAGGGCGAAGGTATAGGGTATCTTCAAACTTTCCTGCTCGGTTTCGTCCGTCAGACA	GG
ZEP-SNP23	7	3,222,996	Ciclev10025089m.g	I					-	5632	C	T	GAATGATTCTTTACATTCAGTTTAACAGCATGTTTCTCAATTAACACTTATAGCTTGTTGGTTGAGCCGTTATAGAGTAGGATTGTAGCCGTTGTGCTTC[C/T]AGGTTCCAACAACGAAAGTGTTGGTTAACTGTCATTTTATCTCAGATAGTCATCTTCTTTACCAATCACCCACCTGCTGTTTCAACTTCAAAGTTTCAGC	GG
ZEP-SNP24	7	3,222,620	Ciclev10025089m.g	3					-	6008	C	A	GGAATTGTACAGCATTTATTGGCGCACTAATGCAAGTCTGATGCCCTGCCTTGTATACAAATGTAGGATCAGAGTAG[C/A]GAGCACAGCACAAGAAATGTCATACTCACACATAGAGCTGCTTTGTTTCAGGGAAAGCTGGAACATTTAATT	GG
NCED-SNP01	2	35,236,903	Ciclev10014639m.g	C	D	433	V	I	+	1297	G	A	AAAGAATGCTAAAGATTCTAACGACATCATCTGGATTGAATCACCGGACACGTTCTGCTTTCACTTGTGGAACGCTTGGGAGGAGCCGGAAACTGATGAA[G/A]TTGTTGTCATTGGATCATGCATGACACCTGCTGACTCAATTTTCAACGAGTGTGACGAGAGTCTGAAGAGCGTTTTATCCGAAATTCGGCTCAATTTAAA	FD
NCED-SNP02	2	35,237,118	Ciclev10014639m.g	C	S	504	A	A	+	1512	G	C	TCCACGCGCCGCCCGATTCTCTCGGAGTCTGATCAAGTGAACTTGGAGGCTGGGATGGTGAATCGCAACAGCCTTGGTAGAAAAACTCAGTTCGCTTATCTAGC[G/C]ATTGCGGAGCCATGGCCTAAAGTTTCAGGTTTTGCTAAAGTGGATCTCTCTTCAGGAGAGGTTAAAAAGTATTTTTACGGCGATAATAAATATGGC	FD

NCED-SNP03	2	35,237,189	Ciclev10014639m.g	C	D	528	Y	F	+	1583	A	T	AGCCTTGGTAGAAAAACTCAGTTCGCTTATCTAGCGATTGCGGAGCCATGGCCTAAAGTTTCAGGTTTTGCTAAAGTGGATCTCTCTTCAGGAGAGGTTAAAAAGT[A/T]TTTTTACGGCGATAATAAATATGGCGGCGAGCCATTTTTCTTGCCAAGAGATGCTAATAATTCTGAAAATGAAGACGATGGTTATATTCTTGCA	FD
NCED-SNP04	2	35,237,648	Ciclev10014639m.g	3					+	2042	C	A	CGGATTCTCCTGGACATTCGTTTAAAAAAAAAAAATTACGTACCTTTTGTTTCTGTTAGGTTTGTGTTTATTACAGGACTTTTAGCTATACTAGTTTTTA[C/A]GGAACCAAGCTTGTAGCTTTTGTCTTGTAGGTAGCATTCGAGCTCAGCTGGTTTCTGGTTTGTTTAATTTGTATTTTTACTGTGTCATGTTCTGTGAAAG	FD
NCED-SNP05	2	35,237,708	Ciclev10014639m.g	3					+	2102	T	C	TTTGTGTTTATTACAGGACTTTTAGCTATACTAGTTTTTACGGAACCAAGCTTGTAGCTTTTGTCTTGTAGGTAGCATTCGAGCTCAGCTGGTTTCTGGT[T/C]TGTTTAATTTGTATTTTTACTGTGTCATGTTCTGTGAAAGGAGGAGACACTCTTTCTCGGTCTCACTGTCTTGAATTGATCAAGAAGATGTCTTCCTCTC	FD
NCED-SNP06	2	35,237,766	Ciclev10014639m.g	3					+	2160	C	G	TTTTGTCTTGTAGGTAGCATTCGAGCTCAGCTGGTTTCTGGTTTGTTTAATTTGTATTTTTACTGTGTCATGTTCTGTGAAGGAGGAGACACTCTTTCT[C/G]GGTCTCACTGTCTTGAATTGATCAAGAAGATGTCTTCCTCTCTTTTACTTGTACTTTCATTCCCTCTTTCCATATTACGCCTTAGAAAATACTAGGCGTC	FD
TCL-SNP01	6	10,485,237									C	G	GAAGGTGATTTGGGTGGAGCTTTGCGCTTTACGCAACCCCTCTTCACTCTCTCCCTCTCCCATCCAAAGATTC[C/G]TTTTTKCTTTCCTTTTTATCTTCTTCTTCCTCTTCCTCTCTCAACACGCACAGTTRCGGATCAACTCTTTGTCCGT	FD
TCL-SNP02	6	10,485,823									A	G	CAATGGAGGTATCCTTGTGGGCCCATGRTGGCGCCTCCTCCTCCATATGTGGAGCACCAGAAGGCGGTTACGATACGGAATGATGTGAATTTGAAGAAGGAGAGTTTGA[A/G]GCTGGAGGCCGATGAGGAGAATCCTGGGAAGCTCTTGGTTTCCTTCACCTTTGATGCTACTGTTGCTGGGAGGTAACTGAAACAAATA	FD
TCL-SNP03	6	10,486,132									G	T	CCATATGGACTTGTTAAGGGATTCTTAAATCCAAATTTCTTTATTGCATAGCGGAATGTCATGTCAACT[G/T]GAATAGTTAACCTAGGAAGTGAGAGCTTGSAGTAGCAATAYAATTGCTTACAAAGAAACTATGTGTACCTTCTATTGTC	FD
TCL-SNP04	6	10,486,162									G	C	ATTGCATAGCGGAATGTCATGTCAACTKGAATAGTTAACCTAGGAAGTGAGAGCTTG[G/C]AGTAGCAATAYAATTGCTTACAAAGAAACTATGTGTACCTTCTATTGTCATTTGTTTGTAGTCCACTGTTAGAGTTAAGCTCGTAGTTTTGTGATATGCTTGGGAATTGT	FD(NCED_1069?)
TCL-SNP05	6	10,486,273									A	T	ATTGCTTACAAAGAAACTATGTGTACCTTCTATTGTCATTTGTTTGTAGTCCACTGTTAGAGTTAAGCTCGTAGTTTTGTGATATGCTTGGGAATTGT[A/T]TGGTTGACATCTTTAGGACCAATTTATGTTTTCAAATATACTTTCTCCAGAATCACTGGAAAGAAGGGGGAAAAAGAAATAAAAGCT	FD
