# Supplementary material for: Allelic composition of carotenoid metabolic genes in 13 founders influences carotenoid composition in juice sac tissues of fruits among Japanese citrus breeding population
Source: PLoS One. 2021 Feb 4;16(2):e0246468. doi: 10.1371/journal.pone.0246468 (PMC7861536; doi:10.1371/journal.pone.0246468)
Supplement: S2 Table — (PDF) [file pone.0246468.s008.pdf]

S2 Table A. Independent alleles of *PSY* in 13 founders

Independent allele	PSY-SNP01	PSY-SNP02	PSY-SNP03	PSY-SNP04	PSY-SNP05	PSY-SNP06	PSY-SNP07	PSY-SNP08	PSY-SNP09	PSY-SNP10	PSY-SNP11	PSY-SNP12
PSY-a	G	A	A	C	T	A	A	A	C	T	A	T
PSY-b	A	G	A	T	T	G	A	G	C	T	G	G
PSY-c	A	G	A	T	T	G	A	G	C	C	G	G
PSY-d	G	A	T	C	C	G	A	A	C	T	A	T
PSY-e	G	A	A	C	T	G	A	A	T	T	A	T
PSY-f	A	G	A	T	T	G	C	A	C	T	G	G
PSY-g	G	A	A	C	T	G	A	A	C	T	A	G

S2 Table B. Independent alleles of *HYb* in 13 founders

Independent allele	HYb-SNP01	HYb-SNP02	HYb-SNP03	HYb-SNP04	HYb-SNP05	HYb-SNP06	HYb-SNP07	HYb-SNP08	HYb-SNP09	HYb-SNP10
HYB-a	A	A	A	G	T	C	C	G	G	C
HYB-b	A	A	G	G	T	T	T	G	G	C
HYB-c	A	A	A	G	T	T	T	G	G	C
HYB-d	A	A	A	G	T	T	T	T	G	C
HYB-e	G	A	G	C	T	T	C	T	A	G
HYB-f	A	A	G	C	G	T	C	T	A	G
HYB-g	A	G	G	G	T	T	T	G	G	C

S2 Table C. Independent alleles of *ZEP* in 13 founders

Independent allele	ZEP-SNP01	ZEP-SNP02	ZEP-SNP03	ZEP-SNP04	ZEP-SNP05	ZEP-SNP06	ZEP-SNP07	ZEP-SNP08	ZEP-SNP09	ZEP-SNP10	ZEP-SNP11	ZEP-SNP12	ZEP-SNP13	ZEP-SNP14	ZEP-SNP15	ZEP-SNP16	ZEP-SNP17	ZEP-SNP18	ZEP-SNP19	ZEP-SNP20	ZEP-SNP21	ZEP-SNP22	ZEP-SNP23	ZEP-SNP24
ZEP-a	A	A	C	C	A	T	A	A	G	C	T	A	A	C	C	C	T	T	G	A	T	C	C	C
ZEP-b	A	A	C	C	A	C	A	A	G	C	T	A	A	C	C	C	C	T	G	A	C	C	C	C
ZEP-c	A	G	C	C	A	T	A	T	T	T	C	G	C	C	T	T	C	C	T	C	C	A	T	A
ZEP-d	T	G	C	C	A	T	A	T	T	T	C	G	C	C	T	T	C	C	T	C	C	A	T	A
ZEP-e	A	A	C	C	A	T	A	A	G	C	T	A	A	C	C	C	T	T	G	A	C	C	C	C
ZEP-f	A	A	C	C	A	T	A	A	G	C	T	A	A	C	C	C	T	T	G	C	T	C	C	C
ZEP-g	T	G	A	C	A	T	A	T	T	T	C	G	C	C	T	T	C	C	T	A	C	A	T	A
ZEP-h	A	G	C	G	A	T	A	T	T	T	C	G	C	T	T	T	C	C	T	C	C	A	T	A
ZEP-i	T	G	C	C	T	T	G	T	T	T	C	G	C	C	T	T	C	C	T	C	C	A	T	A
ZEP-j	T	G	A	C	A	T	A	T	T	T	C	G	C	C	T	T	C	C	T	C	C	A	T	A
ZEP-k	A	G	C	G	T	T	G	T	T	T	C	G	C	C	T	T	C	C	G	C	C	A	C	A

S2 Table D. Independent alleles of *NCED* in 13 founders

Independent allele	NCED-SNP01	NCED-SNP02	NCED-SNP03	NCED-SNP04	NCED-SNP05	NCED-SNP06
NCED-a	A	C	A	A	T	C
NCED-b	G	G	A	C	T	C
NCED-c	G	G	T	C	T	C
NCED-d	A	C	A	A	T	C
NCED-e	A	C	A	A	C	C

S2 Table E. Independent alleles of *TCL* in 13 founders

Independent allele	TCL-SNP01	TCL-SNP02	TCL-SNP03	TCL-SNP04	TCL-SNP05
TCL-a	G	G	T	G	T
TCL-b	C	A	G	C	A
TCL-c	C	A	G	G	A
TCL-d	G	A	T	G	T
