# Supplementary material for: Allelic composition of carotenoid metabolic genes in 13 founders influences carotenoid composition in juice sac tissues of fruits among Japanese citrus breeding population
Source: PLoS One. 2021 Feb 4;16(2):e0246468. doi: 10.1371/journal.pone.0246468 (PMC7861536; doi:10.1371/journal.pone.0246468)
Supplement: S3 Table — (PDF) [file pone.0246468.s009.pdf]

S3 Table. Allelic genotype of 5 target genes for major varieties and cultivars

Code	Name	PSY allele type	HYb allele type	ZEP allele type	NCED allele type	TCL allele type	Total carotenoid (mg/100FWG)	β-Cryptoxanthin (mg/100FWG)	Violaxanthin (mg/100FWG)
1	Dancy tangerine	PSY-b-dc1/PSY-e-dc2	HYb-a-dc1/HYb-d-dc2	ZEP-a-dc1/ZEP-b-dc2	NCED-a-dc1/NCED-c-dc2	TCL-b-dc1/TCL-c-dc2	3.141	1.48	0.679
2	Grapefruit	PSY-c-gf1/PSY-a-gf2	HYb-f-gf1/HYb-b-gf2	ZEP-f-gf1/ZEP-g-gf2	NCED-d-gf1/NCED-a-gf2	TCL-b-gf1/TCL-d-gf2	0	0	0
3	Kishu mikan	PSY-a-ks1/PSY-d-ks2	HYb-g-ks1/HYb-a-ks2	ZEP-a-ks1/ZEP-b-ks2	NCED-b-ksh/NCED-b-ksh	TCL-c-ksh/TCL-c-ksh	4.672	2.205	0.922
4	Buntan pumelo	PSY-c-tb1/PSY-b-tb2	HYb-b-tb1/HYb-a-tb2	ZEP-j-tb1/ZEP-d-tb2	NCED-e-tb1/NCED-a-tb2	TCL-a-tbh/TCL-a-tbh	N/A	N/A	N/A
5	Hassaku	PSY-f-hs1/PSY-a-hs2	HYb-e-hs1/HYb-a-hs2	ZEP-a-hs1/ZEP-h-hs2	NCED-a-hsh/NCED-a-hsh	TCL-a-hsh/TCL-a-hsh	0.394	0.076	0.271
6	Hyuga-natsu	PSY-c-hg1/PSY-g-hg2	HYb-b-hgh/HYb-b-hgh	ZEP-a-hg1/ZEP-k-hg2	NCED-a-hg1/NCED-b-hg2	TCL-c-hg1/TCL-a-hg2	0.048	0.03	0.008
7	Sweet orange	PSY-c-so1/PSY-a-so2	HYb-b-so1/HYb-c-so2	ZEP-a-so1/ZEP-i-so2	NCED-d-so1/NCED-a-so2	TCL-b-so1/TCL-a-so2	1.252	0.13	0.877
8	Iyo	PSY-b-iy1/PSY-d-iy2	HYb-e-iy1/HYb-a-iy2	ZEP-a-iy1/ZEP-d-iy2	NCED-a-iy1/NCED-c-iy2	TCL-b-iy1/TCL-a-iy2	0.607	0.143	0.327
9	Kunenbo mandarin	PSY-a-kb1/PSY-d-kb2	HYb-b-kb1/HYb-a-kb2	ZEP-a-kb1/ZEP-c-kb2	NCED-a-kb1/NCED-b-kb2	TCL-c-kb1/TCL-a-kb2	N/A	N/A	N/A
10	Ponkan mandarin	PSY-b-pk1/PSY-e-pk2	HYb-a-pk1/HYb-d-pk2	ZEP-a-pkh/ZEP-a-pkh	NCED-a-pk1/NCED-c-pk2	TCL-b-pk1/TCL-c-pk2	2.046	0.911	0.559
11	Willowleaf mandarin	PSY-b-wl1/PSY-a-wl2	HYb-b-wlh/HYb-b-wlh	ZEP-a-wlh/ZEP-a-wlh	NCED-b-wlh/NCED-b-wlh	TCL-b-wl1/TCL-c-wl2	1.164	0.623	0.192
12	King mandarin	PSY-b-kg1/PSY-a-kg2	HYb-b-kg1/HYb-c-kg2	ZEP-e-kg1/ZEP-c-kg2	NCED-a-kg1/NCED-c-kg2	TCL-c-kg1/TCL-a-kg2	6.039	0.935	3.906
13	Murcott	PSY-a-mch/PSY-a-mch	HYb-c-mc1/HYb-a-mc2	ZEP-e-mc1/ZEP-b-mc2	NCED-a-mch/NCED-a-mch	TCL-b-mc1/TCL-a-mc2	5.615	2.577	1.696
14	Clementine mandarin	PSY-b-wl1/PSY-a-so2	HYb-b-wlh/HYb-b-so1	ZEP-a-wlh/ZEP-a-so1	NCED-b-wlh/NCED-a-so2	TCL-c-wl2/TCL-b-so1	1.572	0.841	0.398
15	Satsuma mandarin	PSY-d-ks2/PSY-d-kb2	HYb-g-ks1/HYb-a-kb2	ZEP-b-ks2/ZEP-c-kb2	NCED-b-ksh/NCED-b-kb2	TCL-c-ksh/TCL-a-kb2	2.278	1.521	0.357
16	Minneola	PSY-c-gf1/PSY-e-dc2	HYb-b-gf2/HYb-d-dc2	ZEP-g-gf2/ZEP-a-dc1	NCED-a-gf2/NCED-c-dc2	TCL-b-gf1/TCL-c-dc2	N/A	N/A	N/A
17	Seminole	PSY-a-gf2/PSY-e-dc2	HYb-b-gf2/HYb-a-dc1	ZEP-g-gf2/ZEP-a-dc1	NCED-a-gf2/NCED-c-dc2	TCL-b-gf1/TCL-c-dc2	2.118	1.014	0.473
18	Orland	PSY-c-gf1/PSY-e-dc2	HYb-b-gf2/HYb-a-dc1	ZEP-g-gf2/ZEP-b-dc2	NCED-a-gf2/NCED-a-dc1	TCL-d-gf2/TCL-b-dc1	N/A	N/A	N/A
19	Southern yellow	PSY-b-tb2/PSY-d-ks2	HYb-a-tb2/HYb-g-ks1	ZEP-j-tb1/ZEP-b-ks2	NCED-e-tb1/NCED-b-ksh	TCL-a-tbh/TCL-c-ksh	0.184	0.036	0.076
20	Nankou	PSY-d/PSY-b-wl1	HYb-g-ks1/HYb-b	ZEP-c-kb2/ZEP-a	NCED-b/NCED-a-so2	TCL-c-ksh/TCL-b-so1	1.801	1.169	0.279
21	Ariake	PSY-a-so2/PSY-a-so2	HYb-c-so2/HYb-b	ZEP-i-so2/ZEP-a	NCED-d-so1/NCED-a-so2	TCL-b-so1/TCL-c-wl2	3.146	1.411	1.203
22	Sweet spring	PSY-d/PSY-f-hs1	HYb-g-ks1/HYb-a-hs2	ZEP-b-ks2/ZEP-h-hs2	NCED-b/NCED-a-hsh	TCL-c-ksh/TCL-a-hsh	0.864	0.145	0.549
23	Awa orange	PSY-c-hg1/PSY-a-so2	HYb-b-hgh/HYb-b-so1	ZEP-a-hg1/ZEP-a-so1	NCED-b-hg2/NCED-a-so2	TCL-c-hg1/TCL-b-so1	N/A	N/A	N/A
24	JHG	PSY-d/PSY-g-hg2	HYb-g-ks1/HYb-b-hgh	ZEP-b-ks2/ZEP-a-hg1	NCED-b/NCED-a-hg1	TCL-c-ksh/TCL-c-hg1	1.974	0.835	0.584
25	Kiyomi	PSY-d-ks2/PSY-a-so2	HYb-a-kb2/HYb-b-so1	ZEP-b-ks2/ZEP-i-so2	NCED-b/NCED-a-so2	TCL-c-ksh/TCL-b-so1	1.171	0.58	0.351
26	Aki tangor	PSY-d-ks2/PSY-c-so1	HYb-a-kb2/HYb-b-so1	ZEP-b-ks2/ZEP-i-so2	NCED-b/NCED-a-so2	TCL-c-ksh/TCL-b-so1	0.941	0.493	0.343
27	HF9	PSY-d-ks2/PSY-a-so2	HYb-g-ks1/HYb-b-so1	ZEP-b-ks2/ZEP-a-so1	NCED-b/NCED-a-so2	TCL-c-ksh/TCL-a-so2	3.794	1.944	0.602
28	Hayaka	PSY-d/PSY-b-pk1	HYb-a-kb2/HYb-d-pk2	ZEP-b-ks2/ZEP-a-pkh	NCED-b/NCED-a-pk1	TCL-c-ksh/TCL-b-pk1	2.903	1.234	0.586
29	Kankitsu chukanbohonn	PSY-b-kg1/PSY-a-ks1	HYb-c-kg2/HYb-a-ks2	ZEP-e-kg1/ZEP-b-ks2	NCED-a-kg1/NCED-b-ksh	TCL-a-kg2/TCL-c-ksh	4.334	1.06	2.305
30	Kara mandarin	PSY-d-kb2/PSY-b-kg1	HYb-g-ks1/HYb-b-kg1	ZEP-c-kb2/ZEP-e-kg1	NCED-b/NCED-a-kg1	TCL-c-ksh/TCL-c-kg1	3.864	1.922	1.034
31	Encore	PSY-b-kg1/PSY-a-wl2	HYb-c-kg2/HYb-b-wlh	ZEP-e-kg1/ZEP-a-wlh	NCED-a-kg1/NCED-b-wlh	TCL-c-kg1/TCL-c-wl2	3.32	1.345	1.314
32	Wilking	PSY-b-kg1/PSY-a-wl2	HYb-b-kg1/HYb-b-wlh	ZEP-e-kg1/ZEP-a-wlh	NCED-c-kg2/NCED-b-wlh	TCL-a-kg2/TCL-c-wl2	3.414	0.697	1.839
33	Page	PSY-c-gf1/PSY-a-so2	HYb-b-gf2/HYb-b	ZEP-a-dc1/ZEP-a	NCED-a-gf2/NCED-a-so2	TCL-c/TCL-b	2.64	1.183	0.868
34	Robinson	PSY-a-so2/PSY-e-dc2	HYb-b/HYb-a-dc1	ZEP-a/ZEP-b-dc2	NCED-a-so2/NCED-a	TCL-c-wl2/TCL-b-dc1	3.166	1.035	1.361
35	Lee	PSY-b-wl1/PSY-e-dc2	HYb-b/HYb-a-dc1	ZEP-a/ZEP-b-dc2	NCED-a-so2/NCED-a	TCL-c-wl2/TCL-b-dc1	2.413	1.089	0.541
36	Fairchild	PSY-b-wl1/PSY-e-dc2	HYb-b/HYb-a-dc1	ZEP-a/ZEP-g-gf2	NCED-b-wlh/NCED-a	TCL-b-so1/TCL-b-dc1	N/A	N/A	N/A
37	Fortune	PSY-b-wl1/PSY-c-gf1	HYb-b/HYb-a-dc1	ZEP-a/ZEP-b-dc2	NCED-b-wlh/NCED-a	TCL-b-so1/TCL-b-dc1	N/A	N/A	N/A
38	Nova	PSY-b-wl1/PSY-e-dc2	HYb-b/HYb-a-dc1	ZEP-a/ZEP-b-dc2	NCED-b-wlh/NCED-a-dc1	TCL-c-wl2/TCL-b-dc1	N/A	N/A	N/A
39	Osceola tangerine	PSY-a-so2/PSY-e-dc2	HYb-b/HYb-a-dc1	ZEP-a/ZEP-b-dc2	NCED-a-so2/NCED-a	TCL-c-wl2/TCL-b-dc1	2.513	1.312	0.532
40	Seihou	PSY-a-so2/PSY-e-dc2	HYb-b-so1/HYb-b-gf2	ZEP-i-so2/ZEP-g-gf2	NCED-a-so2/NCED-c-dc2	TCL-c/TCL-b	1.049	0.403	0.225
41	Akemi	PSY-a-so2/PSY-e-dc2	HYb-a/HYb-b	ZEP-b-ks2/ZEP-a-dc1	NCED-a-so2/NCED-c-dc2	TCL-b-so1/TCL-b-gf1	0.836	0.472	0.228
42	Okitsu 46 gou	PSY-d/PSY-a-so2	HYb-a-hs2/HYb-c-so2	ZEP-b-ks2/ZEP-i-so2	NCED-a-hsh/NCED-d-so1	TCL-a-hsh/TCL-b-so1	N/A	N/A	N/A
43	Nishinokaori	PSY-d-ks2/PSY-a-so2	HYb-b-so1/HYb-b-so1	ZEP-i-so2/ZEP-a-so1	NCED-b/NCED-a-so2	TCL-b-so1/TCL-b-so1	1.269	0.501	0.458
44	KyOw No.21	PSY-d-ks2/PSY-d-kb2	HYb-b-so1/HYb-g-ks1	ZEP-i-so2/ZEP-b-ks2	NCED-b/NCED-b	TCL-c-ksh/TCL-a-kb2	0.92	0.415	0.211
45	KyOw No.14	PSY-a-so2/PSY-d-ks2	HYb-a-kb2/HYb-g-ks1	ZEP-i-so2/ZEP-b-ks2	NCED-a-so2/NCED-b	TCL-b-so1/TCL-a-kb2	2.212	1.083	0.582
46	Tsunnokaori	PSY-d-ks2/PSY-d	HYb-b-so1/HYb-a-kb2	ZEP-b-ks2/ZEP-b-ks2	NCED-a-so2/NCED-b	TCL-c-ksh/TCL-a-kb2	1.894	1.214	0.253
47	Shiranui	PSY-d-ks2/PSY-b-pk1	HYb-b-so1/HYb-a-pk1	ZEP-i-so2/ZEP-a-pkh	NCED-a-so2/NCED-a-pk1	TCL-c/TCL-b	1.735	0.719	0.563
48	Youkou	PSY-d-ks2/PSY-e-pk2	HYb-b-so1/HYb-a-pk1	ZEP-b-ks2/ZEP-a-pkh	NCED-a-so2/NCED-a-pk1	TCL-b/TCL-c	4.565	1.551	0.959
49	Harumi	PSY-d-ks2/PSY-e-pk2	HYb-b-so1/HYb-d-pk2	ZEP-b-ks2/ZEP-a-pkh	NCED-b/NCED-a-pk1	TCL-b-so1/TCL-b-pk1	2.134	0.84	0.575
50	Setomi	PSY-d-ks2/PSY-b-pk1	HYb-a-kb2/HYb-a-pk1	ZEP-i-so2/ZEP-a-pkh	NCED-a-so2/NCED-a-pk1	TCL-c-ksh/TCL-c-pk2	3.51	1.293	1.509
51	EnOw No.21	PSY-d-ks2/PSY-a-wl2	HYb-b-wlh/HYb-g-ks1	ZEP-e-kg1/ZEP-c-kb2	NCED-a-kg1/NCED-b	TCL-c/TCL-a-kb2	3.972	2.348	0.774
52	Kuchinotsu 39 gou	PSY-b-kg1/PSY-d	HYb-c-kg2/HYb-g-ks1	ZEP-e-kg1/ZEP-c-kb2	NCED-a-kg1/NCED-b	TCL-c/TCL-a-kb2	2.172	0.986	0.788
53	Mihokoru	PSY-d/PSY-a-wl2	HYb-g-ks1/HYb-b-wlh	ZEP-b-ks2/ZEP-e-kg1	NCED-b/NCED-a-kg1	TCL-c-ksh/TCL-c	4.167	1.72	1.537
54	KyEn No.4	PSY-d-ks2/PSY-a-wl2	HYb-a-kb2/HYb-c-kg2	ZEP-i-so2/ZEP-e-kg1	NCED-a/NCED-b	TCL-c-ksh/TCL-c	N/A	N/A	N/A
55	KyEn5	PSY-d/PSY-a-wl2	HYb-a-kb2/HYb-c-kg2	ZEP-i-so2/ZEP-e-kg1	NCED-b/NCED-b-wlh	TCL-c-ksh/TCL-c	1.76	0.587	0.604
56	Tsunonozomi	PSY-d-ks2/PSY-a-wl2	HYb-b-so1/HYb-b-wlh	ZEP-b-ks2/ZEP-e-kg1	NCED-a-so2/NCED-a-kg1	TCL-c-ksh/TCL-c	3.756	1.816	0.99
57	Amaka	PSY-d-ks2/PSY-b-kg1	HYb-b-so1/HYb-c-kg2	ZEP-i-so2/ZEP-e-kg1	NCED-b/NCED-b-wlh	TCL-b-so1/TCL-c	1.857	0.853	0.608
58	Okitsu 45 gou	PSY-d-ks2/PSY-b-kg1	HYb-b-so1/HYb-b	ZEP-b-ks2/ZEP-e-kg1	NCED-a-so2/NCED-b-wlh	TCL-b-so1/TCL-c-wl2	N/A	N/A	N/A
59	Tamami	PSY-d-ks2/PSY-b-kg1	HYb-a-kb2/HYb-b	ZEP-b-ks2/ZEP-e-kg1	NCED-b/NCED-b-wlh	TCL-c-ksh/TCL-c-wl2	2.478	1.464	0.432
60	Benibae	PSY-d-ks2/PSY-a-wl2	HYb-g-ks1/HYb-b-wlh	ZEP-a-so1/ns	NCED-a-so2/NCED-a-kg1	TCL-a-so2/TCL-c	3.443	1.768	0.751
61	Hareyaka	PSY-a-wl2/PSY-e-pk2	HYb-b-wlh/HYb-d-pk2	ZEP-e-kg1/ZEP-a-pkh	NCED-b-wlh/NCED-a-pk1	TCL-c/ns	3.753	1.603	0.983
62	Amakusa	PSY-d-ks2/PSY-c-gf1	HYb-a-kb2/HYb-b	ZEP-i-so2/ZEP-a	NCED-a-so2/NCED-a	TCL-a-kb2/TCL-c	1.081	0.574	0.321
63	Kuchinotsu 38 gou	PSY-d/PSY-a-so2	HYb-g-ks1/HYb-b	ZEP-i-so2/ZEP-a	NCED-b/NCED-a	TCL-a-kb2/TCL-c-wl2	2.407	1.271	0.558
64	Kankitsu chukanbohon r	PSY-e-dc2/PSY-a-ks1	HYb-b/HYb-g-ks1	ZEP-a/ZEP-b	NCED-a/NCED-b-ksh	TCL-c-wl2/TCL-c-ksh	1.39	0.656	0.254
65	E-647	PSY-a-so2/PSY-e-dc2	HYb-b-so1/HYb-b	ZEP-i-so2/ZEP-b-dc2	NCED-a-so2/NCED-a	TCL-b/TCL-c	1.642	0.856	0.471
66	Southern red	PSY-b-kg1/PSY-e-dc2	HYb-g-ks1/HYb-a-dc1	ZEP-e-kg1/ZEP-b-dc2	NCED-a-kg1/NCED-a	TCL-c-wl2/TCL-c	3.666	1.739	0.962
67	Kuchinotsu 28 gou	PSY-d-ks2/PSY-e-dc2	HYb-g-ks1/HYb-a-dc1	ZEP-i-so2/ZEP-b-dc2	NCED-b/NCED-a-dc1	TCL-a-kb2/TCL-c-dc2	N/A	N/A	N/A
68	Haruhi	PSY-a-so2/PSY-a-so2	HYb-c-so2/HYb-b	ZEP-b-ks2/ZEP-a	NCED-d-so1/NCED-b-hg2	TCL-b-so1/TCL-b-so1	1.432	0.794	0.393
69	2700・OIy-25	PSY-d-ks2/PSY-d-iy2	HYb-b-so1/HYb-e-iy1	ZEP-i-so2/ZEP-a-iy1	NCED-a-so2/NCED-c-iy2	TCL-b-so1/TCL-b-iy1	0.636	0.246	0.097
70	No.1408	PSY-d-ks2/PSY-d-ks2	HYb-g-ks1/HYb-e-iy1	ZEP-e-kg1/ZEP-d-iy2	NCED-b/NCED-a	TCL-c/TCL-b	1.753	0.843	0.333
71	Kuchinotsu 18 gou	PSY-d-ks2/PSY-b-kg1	HYb-b-so1/HYb-c-kg2	ZEP-i-so2/ZEP-e-kg1	NCED-b/NCED-b-wlh	TCL-a-kb2/TCL-c	1.206	0.767	0.201
72	Kuchinotsu 35 gou	PSY-d-ks2/PSY-b-kg1	HYb-g-ks1/HYb-b-wlh	ZEP-b-ks2/ZEP-a-wlh	NCED-b/NCED-a-kg1	TCL-a-kb2/TCL-c	2.482	1.143	0.615
73	Kanpei	PSY-d-ks2/PSY-e-pk2	HYb-b-so1/HYb-a-pk1	ZEP-i-so2/ZEP-a-pkh	NCED-a-so2/NCED-c-pk2	TCL-b-so1/TCL-c-pk2	3.226	1.478	0.927
74	Okitsu 57 gou	PSY-d/PSY-e-pk2	HYb-c-so2/HYb-d-pk2	ZEP-b-ks2/ZEP-a-pkh	NCED-d-so1/NCED-a-pk1	TCL-b-so1/TCL-b	4.377	1.81	0.956
75	Asumi	PSY-d/PSY-e-pk2	HYb-a-hs2/HYb-d-pk2	ZEP-b-ks2/ZEP-a-pkh	NCED-a-hsh/NCED-a-pk1	TCL-b-so1/TCL-b	3.179	1.661	0.752
76	Asuki	PSY-d/PSY-e-pk2	HYb-a-hs2/HYb-b-so1	ZEP-i-so2/ZEP-b-ks2	NCED-a-hsh/NCED-a-pk1	TCL-a-hsh/TCL-b	2.701	1.313	0.873
77	Seinannohikari	PSY-d-ks2/PSY-d-ks2	HYb-g-ks1/HYb-a-pk1	ZEP-c-kb2/ZEP-a-pkh	NCED-b/NCED-a	TCL-a-kb2/TCL-c	5.035	2.536	1.263
78	Kuchinotsu 27 gou	PSY-d-ks2/PSY-d-ks2	HYb-b-wlh/HYb-a-pk1	ZEP-c-kb2/ZEP-b-ks2	NCED-a-kg1/NCED-a	TCL-c/TCL-b	2.874	1.525	0.578
79	Kuchinotsu 33 gou	PSY-d-ks2/PSY-b-kg1	HYb-a-kb2/HYb-c-kg2	ZEP-i-so2/ZEP-e-kg1	NCED-a/NCED-b	TCL-a-kb2/TCL-a	1.612	0.475	1.055
80	Tsunokagayaki	PSY-a-so2/PSY-a-wl2	HYb-a-kb2/HYb-b-wlh	ZEP-i-so2/ZEP-e-kg1	NCED-a/NCED-b	TCL-b-so1/TCL-c	4.617	2.18	1.236
81	Setoka	PSY-a-wl2/PSY-a-mch	HYb-a-kb2/HYb-a-mc2	ZEP-e-kg1/ZEP-e-mc1	NCED-a/NCED-a-mch	TCL-c/TCL-b-mc1	4.161	1.509	1.534
82	Kuchinotsu 36 gou	PSY-d-ks2/PSY-a-mch	HYb-b/HYb-a-mc2	ZEP-i-so2/ZEP-e-mc1	NCED-a/NCED-a-mch	TCL-c/TCL-b-mc1	3.332	1.47	1.139
83	Reiou	PSY-d/PSY-a-mch	ns/ns	ns/ns	NCED-b/NCED-a-mch	TCL-c/TCL-b-mc1	1.694	1.021	0.397
84	Ehime kashi 28 gou	PSY-d/PSY-c-gf1	HYb-g-ks1/HYb-b	ZEP-a/ZEP-a	NCED-a-so2/NCED-a	TCL-b-so1/TCL-a-kb2	1.247	0.666	0.416
85	Kuchinotsu 49 gou	PSY-a-so2/PSY-d-ks2	HYb-g-ks1/HYb-g-ks1	ZEP-i-so2/ZEP-e-kg1	NCED-b/NCED-a	TCL-c-wl2/TCL-b	3.134	1.705	0.594
86	Okitstu 56 gou	PSY-d-ks2/PSY-a-ks1	HYb-b/HYb-g-ks1	ZEP-b-ks2/ZEP-a	NCED-b-wlh/NCED-b-ksh	TCL-b-so1/TCL-c	2.111	1.152	0.406
87	No.1011	PSY-b-wl1/PSY-d-ks2	HYb-b/HYb-b-so1	ZEP-c-kb2/ZEP-a	NCED-a-so2/NCED-c-iy2	TCL-b-so1/TCL-b	0.918	0.56	0.095
88	Kuchinotsu 51 gou	PSY-d/PSY-d-ks2	ns/ns	ns/ZEP-b-ks2	NCED-b/NCED-a	TCL-c/TCL-c	2.073	1.016	0.657
89	Harehime	PSY-e-dc2/PSY-d	HYb-b/HYb-a-kb2	ZEP-b-dc2/ZEP-c-kb2	NCED-a/NCED-b	TCL-c/TCL-a-kb2	1.24	0.711	0.152
90	Okitsu 63 gou	PSY-e-dc2/PSY-b-pk1	HYb-b/HYb-d-pk2	ZEP-i-so2/ZEP-a-pkh	NCED-a/NCED-a-pk1	TCL-b/TCL-c	1.998	0.74	0.718
91	Kuchinotsu 52 gou	PSY-a-so2/PSY-a	HYb-b-wlh/HYb-b	ZEP-e-kg1/ZEP-a	NCED-b/NCED-a-so2	TCL-c/TCL-c-wl2	4.49	2.307	1.376
92	Mihaya	PSY-d-ks2/PSY-d-ks2	HYb-b/HYb-g-ks1	ZEP-e-kg1/ZEP-d	NCED-a/NCED-a	TCL-c/TCL-b	2.437	1.477	0.482
93	Kuchinotsu 54 gou	PSY-d-ks2/PSY-a-ks1	HYb-a-mc2/HYb-a-ks2	ZEP-e-mc1/ZEP-e-kg1	NCED-a/NCED-a-kg1	TCL-b-mc1/TCL-c-ksh	4.493	1.567	1.734
94	080716	PSY-d/PSY-d-ks2	ns/ns	ZEP-a/ZEP-a-pkh	NCED-a/NCED-b	TCL-b-so1/TCL-c	N/A	N/A	N/A
95	Okitsu 67 gou	PSY-d/PSY-a-ks1	HYb-a-kb2/HYb-b	ZEP-b-dc2/ZEP-a	NCED-a/NCED-b	TCL-a-kb2/TCL-b-so1	N/A	N/A	N/A
