# Supplementary material for: Allelic composition of carotenoid metabolic genes in 13 founders influences carotenoid composition in juice sac tissues of fruits among Japanese citrus breeding population
Source: PLoS One. 2021 Feb 4;16(2):e0246468. doi: 10.1371/journal.pone.0246468 (PMC7861536; doi:10.1371/journal.pone.0246468)
Supplement: Attachment — Submitted filename: Letter for Editor (BCR)(R3.1.6).docx [file pone.0246468.s010.docx]

Dear Editor, Dr. Dr. Chunxian Chen

We are appreciated with the meaningful comments and suggestions of reviewers, which are helpful to improve the quality of our manuscript. Please find attached a new version in which we have tried to address the comments raised by the reviewers. We hope it will be acceptable for publication in PLOS One. We describe our responses against each comment in below. All modifications in new manuscript are written in red characters.

Yours sincerely

Dr. Takehiko Shimada

NARO Institute of Fruit and Tea Tree Science

Department of Citriculture,

Okitsu, Shimizu, Shizuoka 424-0292, Japan

Tel: +81-543-69-7100, Fax: +81-543-69-2115

E-mail: [tshimada@affrc.go.jp](mailto:tshimada@affrc.go.jp)

Response to comment and suggestion

The paper was to identify alleles of carotenoid biosynthetic genes using citrus breeding parents and populations using the SureSelect target enrichment method. The results are of great value for understanding the diversity of carotenoid gene expressions and compositions. The paper was well written. The authors need attentions to some editorial issues in the paper.

Introduction:

Some early relevant works on citrus carotenoid biosynthetic genes/alleles and the pigment compositions should be cited, for example, those from Ollitrault's group (e.g., Fanciullino et al. 2007) and Dr. Gmitter's group (e.g., Chen et al. 2010).

Answer) The recommended reports are added in new manuscript.

Abstract and main text:

Abbreviations are needed only when they are used afterwards (no need of abbreviations otherwise). This rule is also applied to the main text.

Answer) Unnecessary abbreviations are deleted in new manuscript according to editor’s suggestion.

Figures and tables:

They should be understood well without reference to the main text. Some legends and captions should have a bit more illustration. Likewise, abbreviations should be redefined provided that they are used there afterwards.

Answer) Legends are modified for better understanding and attention is paid to the description method of the abbreviation in the legends.
